# Effects of Sterols on the Interaction of SDS, Benzalkonium Chloride, and A Novel Compound, Kor105, with Membranes

**DOI:** 10.3390/biom9100627

**Published:** 2019-10-18

**Authors:** Irene Jiménez-Munguía, Pavel E. Volynsky, Oleg V. Batishchev, Sergey A. Akimov, Galina A. Korshunova, Ekaterina A. Smirnova, Dmitry A. Knorre, Sviatoslav S. Sokolov, Fedor F. Severin

**Affiliations:** 1 Department of Engineering of Technological Equipment, National University of Science and Technology “MISiS”, 4 Leninskiy Prospect, Moscow 119049, Russia; 2 Laboratory of Biomolecular Modeling, Shemyakin-Ovchinnikov Institute of Bioorganic Chemistry, Russian Academy of Sciences, 16/10 Miklukho-Maklaya Str., Moscow 117997, Russia; 3 Laboratory of Bioelectrochemistry, A.N. Frumkin Institute of Physical Chemistry and Electrochemistry, Russian Academy of Sciences, 31/4 Leninskiy Prospekt, Moscow 119071, Russia; 4 Department of Physics of Living Systems, Moscow Institute of Physics and Technology, 9 Institutskiy Lane, Dolgoprudniy, Moscow Region 141700, Russia; 5 Department of Theoretical Physics and Quantum Technologies, National University of Science and Technology “MISiS”, 4 Leninskiy Prospect, Moscow 119049, Russia; 6 Department of Protein Chemistry, Belozersky Institute of Physico-Chemical Biology, Lomonosov Moscow State University, 1–40 Leninskie Gory, Moscow 119991, Russia; 7 Department of Molecular Energetics of Microorganisms, Belozersky Institute of Physico-Chemical Biology, Lomonosov Moscow State University, 1–40 Leninskie Gory, Moscow 119991, Russia; 8 Institute of Molecular Medicine, Sechenov First Moscow State Medical University, 8/2 Trubetskaya Str., Moscow 119991, Russia

**Keywords:** ionic surfactant, sterol, lipid membrane, yeast, inner membrane field compensation, molecular dynamics

## Abstract

Sterols change the biophysical properties of lipid membranes. Here, we analyzed how sterols affect the activity of widely used antimicrobial membrane-active compounds, sodium dodecyl sulfate (SDS) and benzalkonium chloride (BAC). We also tested a novel benzalkonium-like substance, Kor105. Our data suggest that benzalkonium and Kor105 disturb the ordering of the membrane lipid packaging, and this disturbance is dampened by cholesterol. The disturbance induced by Kor105 is stronger than that induced by BAC because of the higher rigidity of the Kor105 molecule due to a shorter linker between the phenyl group and quaternary nitrogen. On the contrary, individual SDS molecules do not cause the disturbance. Thus, in the tested range of concentrations, SDS–membrane interaction is not influenced by cholesterol. To study how sterols influence the biological effects of these chemicals, we used yeast strains lacking Lam1–4 proteins. These proteins transport sterols from the plasma membrane into the endoplasmic reticulum. We found that the mutants are resistant to BAC and Kor105 but hypersensitive to SDS. Together, our findings show that sterols influence the interaction of SDS versus benzalkonium chloride and Kor105 with the membranes in a completely different manner.

## 1. Introduction

Different organisms significantly vary in their sterol content. Indeed, while bacterial cells are usually devoid of sterols, in eukaryotes, sterols represent up to 40 mol.% of total lipids in the plasma membrane (PM) [1] (see also References [2,3]). Moreover, eukaryotes are able to adjust the sterol content of the PM in response to environmental challenges (see Reference [4]). For instance, in response to moderate hyperosmotic stress, yeast cells decrease the sterol levels in their PMs [5]. Many antimicrobial compounds specifically affect only sterol-rich membranes. For example, steroid glycoside digitonin can permeabilize only sterol-rich membranes [6]. Additionally, some polypeptide toxins are specific to sterol-containing membranes [7].

Sterols significantly change the physico-chemical properties of the membrane [8] and affect the mode of membrane interaction with almost every low-molecular-weight compound. For example, sterols increase the resistance of lipid bilayers to detergents [9,10,11,12]. However, the mechanisms of surfactant interactions with membranes with different levels of sterols are still unclear. In addition, many surfactants are used as unspecific biocides for food preservation and decontamination of surfaces, and their wide usage promotes the emergence of microbial resistance to these compounds [13]. Moreover, surfactants are used against fungal contamination [14], while the fungi are highly diverse based on the type [15] and, presumably, content of their sterols. Thus, the understanding of the interaction of specific surfactants with membranes can reveal possible mechanisms contributing to a resistance to these compounds.

Importantly, while biological membranes have a negative surface electric charge, concentrated in either the inner (mammalian or yeast cells) or the outer (bacterial cells) membrane leaflet [16,17,18], sterols are not charged. Therefore, sterols may decrease the electric potential by diluting charged phospholipids and, in this way, decrease the toxicity of cationic surfactants. If so, membrane-incorporated sterols are likely to have an opposite effect on the interaction of sodium dodecyl sulfate (SDS) (negatively charged) and benzalkonium (positively charged) with the membranes. Moreover, the lipid heads of the outer leaflet lipids of mammalian and fungal PMs are mostly dipolar, with spatially separated positive and negative charges. In such membranes, sterols may influence the accessibility of electric charges of the lipid heads to charged surfactants added to the aqueous phase. As positively and negatively charged lipid groups are located at different depths in the lipid monolayer, the accessibility of the group is expected to vary differently upon the changes in sterol concentration.

Therefore, one might expect the sterol content to differentially affect the mode of surfactant–membrane interaction depending on a particular surfactant. Thus, we studied how sterols affect the interaction of the membranes with the two possibly most well-known surfactants, SDS and benzalkonium chloride (BAC). We tested this assumption using several approaches.

**Figure 1:**
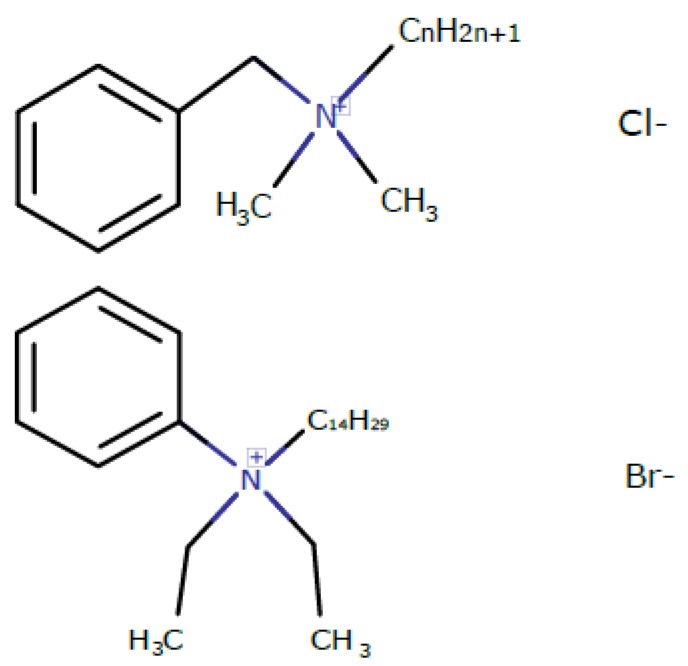
Chemical structures of benzalkonium chloride (BAC; top) and Kor105 (bottom).

Firstly, we added SDS and BAC to planar bilayer lipid membranes (BLMs) and measured how these substances affected the boundary potential of the membrane. We also tested a novel benzalkonium-like compound, Kor105. In this compound, the aromatic ring is directly linked to nitrogen (Figure 1), which makes the ring less mobile. This means that Kor105 might be more active in terms of disturbing the packaging of the membrane lipid molecules. The lipid we used to produce the membrane, 1,2-dioleoyl-*sn*-glycero-3-phosphocholine (DOPC), contains one positively and one negatively charged group. Although the DOPC molecule is electrically neutral at neutral pH, its positively (choline) and negatively (phosphate) charged groups are spatially separated, and the phosphate group is buried relatively deep within the lipid monolayer, as shown for other phosphocholines [19]. For this reason, we expected that such a membrane would differently interact with anionic versus cationic surfactants.

We also modeled the effect of cholesterol on the interaction of benzalkonium, Kor105, and SDS ions with the membrane using molecular dynamics (MD) simulations. As our results predicted that the sterol content of the PM may strongly influence the effects of benzalkonium, Kor105, and SDS on cell physiology, we performed a series of experiments on yeast cells harboring mutations in *Lam* (also called *Ysp* or *Ltc*) genes, which encode PM sterol transporters [4,20,21,22]. Recently it was shown that, in mammals, Lam protein homologs facilitate cholesterol transport from plasma membrane to endoplasmic reticulum (ER) [23]. Thus, it can be expected that deletions of *Lam* genes increase the PM ergosterol content. Our experiments on yeast cells, in agreement with those on BLMs and computer modeling, showed that the effects of sterols on the interaction of SDS versus the positively charged surfactants with the membranes are fundamentally different.

## 2. Materials and Methods

### 2.1. Synthesis of Kor105 (Diethyl (Tetradecyl) Phenyl Ammonium Bromide)

All reagents were purchased from commercial sources and used without further purification: di(ethyl)aniline (Acros Organics, Geel, Belgium), 1-bromtetradecan (Koch-Light Laboratories Ltd., Gauteng, South Africa), silica gel 60 F254 (Merck, Kenilworth, NJ, USA).

The mass spectra of the synthesis products were measured using the UPLC/MS/MS system consisting of an Acquity UPLC system (Waters, Milford, MA, USA) and a tandem quadrupole (TQD) mass spectrometer (Waters, Milford, MA, USA). Analytical HPLC was performed with an Agilent 1100 equipped with a 4.6 × 150 mm column Luna 5 µm C18 (flow rate, 1.5 mL/min; 250 °C). Thin-layer chromatography (TLC) was carried out on 0.2-mm precoated plates of silica gel 60 F254.

A solution of 149 mg of di(ethyl)aniline in 0.5 mL of ethanol (0.1 mM) was added to a solution of 277 mg of 1-bromtetradecane in 0.5 mL of ethanol (0.1 mM) with stirring at room temperature for 20 h and then for 2 h at 75 °C. Monitoring of the process was carried out with the help of ultraviolet (UV) absorbance and Dragendorf reagent on TLC plates. The reaction mixture was evaporated in vacuum to dryness, and the residue was dissolved in a minimal quantity of dichloromethane and precipitated by *n*-hexane until precipitation was complete. After cooling, the precipitate was separated by decantation and treated twice to obtain crude target diethyl (tetradecyl) phenyl ammonium bromide. The substance was dried in vacuum and subjected to column chromatography on silica gel using chloroform/methanol 3:1 as the eluent system. The fractions with the same values of retention factor (Rf) were collected and evaporated in vacuum. Yield of pale solid 148 mg (35%) TLC:Rf (chloroform/methanol 3:1) 0.79; LC–MS *m*/*z* calculated for C_24_H_44_N 346.5, found 346.5.

### 2.2. Study of the Effect of BAC and Kor105 on Model Membranes

Model BLMs were formed by the Muller–Rudin method [24] on a 1-mm-wide orifice in a vertical Teflon film separating two compartments of a specially designed chamber. Working buffer was prepared using twice distilled water with 20 mM KCl and 2 mM of 2-[4-(2-hydroxyethyl)piperazin-1-yl]ethanesulfonic acid (HEPES) at pH 7.0, which was adjusted by HCl. Each compartment was filled with 2 mL of working buffer and continuously stirred with a magnetic stirrer. Stock solutions of BAC and Kor105 were prepared to a final concentration of 5 mM and were appropriately diluted prior to addition to the system. Electrical measurements were performed using Ag/AgCl electrodes placed in each compartment. The concentration of lipid solution used for membrane formation was 15 mg/mL in decane (Sigma, St. Louis, MO, USA). Membranes were formed either from DOPC or from a 3:2 mixture of DOPC and cholesterol (Chol) (both from Avanti Polar Lipids Inc., Alabaster, AL, USA). The lipid solution was painted over the orifice. After several seconds, the bilayer was spontaneously formed. The formation of the bilayer was registered as an increase in electric capacitance between the electrodes located in two different compartments of the chamber, separated by the membrane.

**Figure 2:**
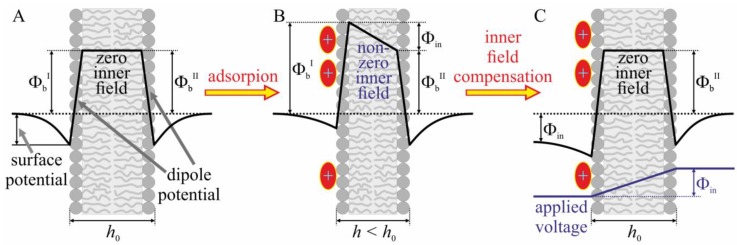
Illustration of the method of inner membrane field compensation; **A**—initial distribution of the electric potential in the vicinity of the membrane. The boundary potential Φ_b_ is a sum of the dipole potential and surface potential measured in the diffuse part of electrical double layer. Due to the symmetry, the electric potential is constant inside the membrane, i.e., the inner electric field is equal to zero. The membrane thickness is denoted as *h*_0_. **B**—Asymmetric (one-side) adsorption of charged molecules to the membrane yields asymmetric distribution of the electric potential: Φ_in_ now drops inside the membrane. Non-zero inner electric field arises, leading to a decrease in membrane thickness, *h* < *h*_0_, and an increase in membrane electric capacitance, due to the electrostriction. **C**—Application of the external voltage equal to Φ_in_ compensates for the inner membrane field. The equality of the applied voltage to Φ_in_ can be controlled by measuring the dependence of membrane electric capacitance on the applied voltage. When the capacitance is minimal (the membrane thickness is maximal and equal to *h*_0_), the inner electric field is equal to zero (as in panel A) and, thus, the applied voltage is equal to Φ_in_.

The boundary potential difference (∆Φ_b_) was measured using the inner field compensation (IFC) method, which utilizes the second harmonics of the capacitance current for capacitance minimization [25]. The method allows the measurement of the transmembrane voltage arising from asymmetric (one-side) adsorption of charged molecules onto the membrane [26,27,28] (Figure 2). The boundary potential difference was measured for different bulk concentrations of hydrophobic ions added to one of two compartments of the experimental chamber.

Boundary potential at one side of the membrane reflects the potential difference between a point in the bulk water solution far from the membrane and a point at the boundary between polar and nonpolar parts of the membrane. Therefore, the boundary potential is a sum of the surface potential measured in the diffuse part of electrical double layer and dipole potential arising from mutual orientation of water dipoles in proximity of the membrane and dipoles of polar heads of lipids. According to the Gouy–Chapman model [29,30], surface potential of the lipid bilayer depends on its surface charge density and ionic strength of electrolyte solution, and mainly reflects the adsorption of charged molecules at the interface between membrane and water solution.

Initially, the distribution of the electric potential is symmetric, the boundary potentials of left (Φ_b_^I^) and right (Φ_b_^II^) monolayers are equal to each other, and the electric field inside the membrane is equal to zero (Figure 2A). When charged molecules are asymmetrically adsorbed onto the membrane (to the left monolayer in Figure 2B) the distribution of the electric potential also becomes asymmetric. The boundary potential of the opposite monolayer, Φ_b_^II^, does not change, while the boundary potential of the left monolayer changes proportionally to the electric charge and surface density of the adsorbed molecules. The difference between the boundary potentials of the left and right monolayers, Φ_in_, now drops inside the membrane. The non-zero inner electric field leads to electrostriction; the membrane thickness decreases, or, equivalently, its electric capacitance increases (Figure 2B). Externally applied voltage can compensate for the inner membrane electric field, if it is exactly equal to Φ_in_ (Figure 2C). Application of the external voltage leads to change of the membrane capacitance as the voltage alters the inner membrane electric field. When the capacitance is minimal (the membrane thickness is maximal), the inner field equals to zero (Figure 2A,C). This occurs when the applied voltage is equal to Φ_in_. Thus, measuring the dependence of the membrane electric capacitance on the applied voltage allows obtaining Φ_in_—the change in boundary potential caused by asymmetric adsorption of charged molecules.

If an adsorbed charged molecule can penetrate between lipid polar heads, thus influencing the dipole potential of the membrane, the IFC method cannot distinguish between changes in surface or dipole parts of the boundary potential. Thus, IFC cannot give information about the position of the molecule in the membrane. To solve this problem, in addition to IFC, we used an alternative method for determining the boundary potential of BLM after the addition of hydrophobic ions. This method measures the influence of the tested substances on the energy barrier for the transmembrane transport of the ionophore nonactin [31], as determined by changes in the membrane conductance in the presence of nonactin. This barrier is mainly determined by the membrane dipole potential and independent on the surface charge density; thus, the latter technique is sensitive to the penetration of substances through the membrane. Comparison of IFC and nonactin conductance allowed evaluation of the penetration of hydrophobic ions into the BLM.

### 2.3. MD Modeling of Membranes with Incorporated Hydrophobic Ions

MD simulations were performed with Gromacs 5.12 using the CHARMM36 [32] force field and transferable intermolecular potential with 3 points (TIP3P) water model [33]. The molecular topologies of BAC and Kor105 were created using lipid templates for alkyl chain and protein templates for the charged heads of ions. We used the charge shift 0.4 *e*^−^ for the bonds N–aliphatic C and 0 *e*^−^ for the bond N–aromatic C. Membranes of 12 different compositions were modeled: (1) 200 DOPC, one Kor105 (lowKorDOPC); (2) 120 DOPC, 80 Chol, one Kor105 (lowKorCholDOPC); (3) 202 DOPC, 86 Kor105 (highKorDOPC); (4) 122 DOPC, 80 Chol, 86 Kor105 (highKorCholDOPC); (5) 200 DOPC, one BAC (lowBacDOPC); (6) 120 DOPC, 80 Chol, one BAC lowBacCholDOPC; (7) 202 DOPC, 86 BAC (highBacDOPC); (8) 122 DOPC, 80 Chol, 86 BAC (highBacCholDOPC); (9) 200 DOPC, one SDS (lowSDSDOPC); (10) 120 DOPC, 80 Chol, one SDS (lowSDSCholDOPC); (11) 202 DOPC, 86 SDS (highSDSDOPC); (12) 120 DOPC, 80 Chol, 86 SDS (highSDSCholDOPC). A single hydrophobic ion molecule was placed into the upper monolayer of the horizontal membrane (systems lowKorDOPC, lowKorCholDOPC, lowBacDOPC, lowBacCholDOPC). In the membranes containing many molecules of hydrophobic ions (highKorDOPC, highKorCholDOPC, highBacDOPC, highBacCholDOPC), the molecules were distributed evenly between the monolayers. At least 10,000 water molecules were added to each system, and they were equilibrated by 1000 steps of steepest descent energy minimization followed by 1-ns MD simulation with constant temperature (295 K, Nose–Hoover thermostat [34]) and semi-isotropic pressure (1 bar, Berendsen barostat [35]). The bilayer was then analyzed through a 500-ns MD run at constant temperature and pressure. The temperature was maintained at 295 K with the Nose–Hoover thermostat. The pressure was controlled semi-isotropically with the Parrinello–Rahman barostat [36]. The MD integration step was 0.002 ps; van der Waals interactions were truncated using a 1.4-nm spherical cut-off. Electrostatic effects were treated using a particle-mesh Ewald scheme. For analysis, we used the last 100 ns of the trajectories.

We determined the dependence of the average electrical charge density on the *z* coordinate along the *Oz* axis, perpendicular to the average membrane plane. The bilayer was decomposed into parallel layers *S_z_* (*z* − *dz*; *z* + *dz*). The local charge was averaged using the Gromacs utility over the last 100 ns of the simulation. The profiles were analyzed within 3.5 nm from the membrane center with a step of 0.1 nm.

### 2.4. Yeast Strains and Growth Analysis

We used the standard laboratory *W303-1A Saccharomyces cerevisiae* yeast strain and its derivatives that were produced by transformation of the gene cassette as described in Reference [37]. Each deletion strain was confirmed by PCR with independent sets of primers. To analyze the yeast growth, we took exponentially grown yeast cultures and adjusted the cell density to an optical density (OD) of 0.2 (SpectrostarNano, *λ* = 550 nm). Growth curves were obtained by measuring the OD of yeast suspension cultures grown in a SpectrostarNano plate spectrophotometer with the following settings: temperature, 30 °C; plate shaking, 500 rpm; OD measurements, every 5 min. To compare the relative growth rates, we analyzed the increase in OD during the first 9 h of growth for the wild-type and mutant strains. We excluded from the analysis the data of the first 30 min of the experiment.

## 3. Results and Discussion

### 3.1. Effect of Hydrophobic Ions on Model Membranes

To test the effect of sterol inclusion within the membrane, we studied the adsorption of hydrophobic ions added asymmetrically (to one side) onto model lipid membranes of different compositions by measuring differences in the boundary potential, ∆Φ_b_. The boundary potential difference was measured using the inner field compensation method. The method allows the measurement of the transmembrane voltage arising from asymmetric adsorption of charged molecules onto the membrane [26,27,28]. However, IFC cannot give information about the position of the molecule in the membrane. Thus, apart from IFC, we used an alternative method for determining the boundary potential of BLM after the addition of hydrophobic ions. This method measures the influence of the tested substances on the energy barrier for the transmembrane transport of the ionophore nonactin [31]. This barrier is mainly determined by the membrane dipole potential and independent of the surface charge density; thus, the latter technique is sensitive to the penetration of substances into the membrane. Comparison of the IFC and nonactin conductance allowed evaluation of the penetration of hydrophobic ions into the BLM.

**Figure 3:**
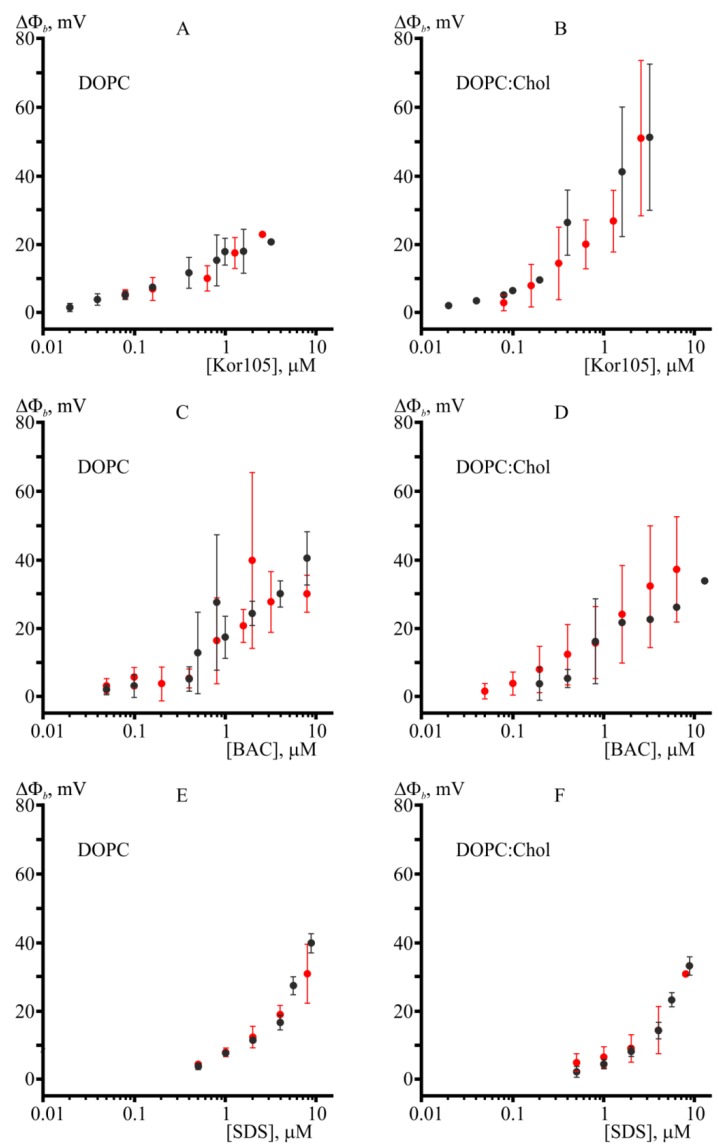
BAC, Kor105, and sodium dodecyl sulfate (SDS) adsorption onto and penetration into bilayer lipid membranes (BLMs). Panels **A** and **B** show the boundary potential difference measured after the addition of increasing concentrations of Kor105. Panels **C** and **D** show the boundary potential difference measured after the addition of increasing concentrations of BAC. Panels **E** and **F** show the sign-inversed boundary potential difference measured after the addition of increasing concentrations of SDS. Membranes formed from 1,2-dioleoyl-*sn*-glycero-3-phosphocholine (DOPC) correspond to panels **A**, **C**, and **E**. Membranes formed from DOPC/cholesterol (Chol) 3:2 correspond to panels **B**, **D**, and **F**. Boundary potential differences (∆Φ_b_) were measured using the inner field compensation (IFC) method (black solid circles) and nonactin conductance (red solid circles).

Changes in the boundary potential were monitored after the addition of increasing concentrations of hydrophobic ions (Figure 3). The potential stabilized approximately 5 min after the addition. A positive change in the potential corresponds to the binding of positively charged molecules or dipoles oriented with their positive pole toward the membrane interior. To evaluate the possible penetration of hydrophobic ions into lipid membranes, we measured the effect of increasing concentrations of these compounds on the conductance of lipid membranes induced by nonactin. Each point in the plots of Figure 3 was obtained by averaging the results of 3–6 experiments. The error bars in the plots represent the standard deviation. If the error bars were smaller than the size of the circle in the plot, the error bars were not shown.

In the case of BAC adsorption on DOPC membranes for concentrations slightly above 1 μM, the membranes became very unstable, which was observed as a substantial decrease in the average lifetime of BLM before its rupture. Most likely, the overall instability of the membranes was responsible for large error bars in the plot (Figure 3C). Interestingly, the presence of cholesterol in the membrane almost eliminated such effects on ∆Φ_b_ induced by BAC adsorption (Figure 3D). This result means that cholesterol increases the membrane ordering, suppressing the disturbance of lipid packing resulting from incorporation of BAC into the lipid membrane. The hypothesized membrane ordering upon cholesterol addition is in accordance with the data obtained from molecular modeling, as described in the next section. Kor105 showed adsorption in a concentration-dependent manner for both membrane compositions used in this study (Figure 3A,B). The presence of 40 mol.% cholesterol in the membrane significantly enhanced the adsorption of Kor105 at concentrations above 0.4 μM, as demonstrated by higher positive values of the boundary potential difference (Figure 3B). Most likely, the difference in adsorption of BAC and Kor105 is due to the fact that in Kor105 the aromatic ring is directly linked to nitrogen, which makes the ring less mobile, as compared to BAC (Figure 1).

To evaluate the penetration of BAC, Kor105, and SDS into and through lipid membranes formed from DOPC and the DOPC/Chol 3:2 mixture, we measured the effect of increasing concentrations of these compounds on the conductance of lipid membranes induced by nonactin. We observed that Kor105, BAC, and SDS did not manifest penetration through the BLMs for both membrane compositions (Figure 3). For all tested compounds, the boundary potentials determined by IFC and nonactin conductance were almost the same within the experimental error, meaning that Kor105, BAC, and SDS neither pass through the membrane nor crucially disturb the monolayer dipoles. In the case of Kor105, the absence of cholesterol significantly decreased adsorption onto the membrane, in contrast to BAC and SDS, where cholesterol addition had almost no effect on adsorption determined by both IFC and nonactin conductance methods. In the presence of cholesterol, all three tested ions yielded almost the same magnitude of the boundary potential difference within the experimental error.

**Figure 4:**
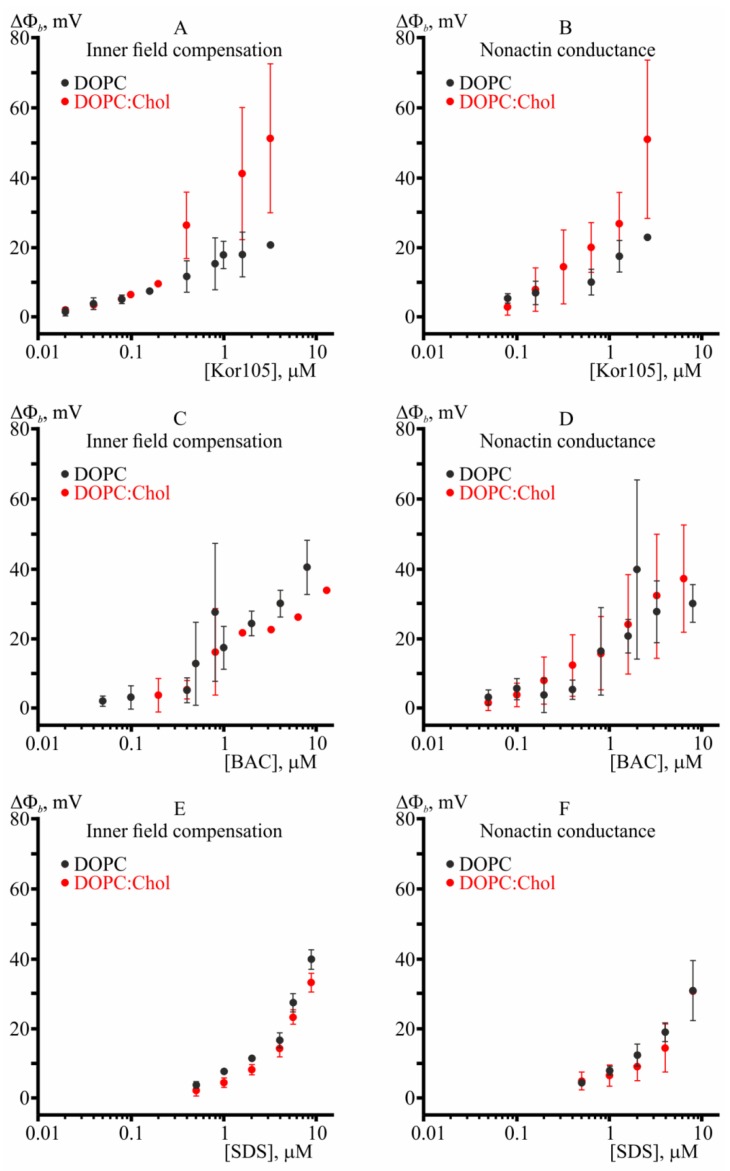
Kor105, BAC, and SDS interaction with BLMs. Panels **A** and **B** show the boundary potential difference measured after the addition of increasing concentrations of Kor105. Panels **C** and **D** show the boundary potential difference measured after the addition of increasing concentrations of BAC. Panels **E** and **F** show the sign-inversed boundary potential difference measured after the addition of increasing concentrations of SDS. The boundary potential measured using the IFC method is demonstrated in panels **A**, **C**, and **E**. The boundary potential measured using the nonactin conductance method is demonstrated in panels **B**, **D**, and **F**. The data obtained on DOPC membranes are shown as black solid circles; the data obtained on DOPC/Chol 3:2 membranes are shown as red solid circles. The data points are the same, as presented in Figure 3, but combined in order to present DOPC vs. DOPC/Chol curves in the same graph.

In Figure 3, it was essential to demonstrate boundary potential curves obtained by IFC and nonactin conductance methods on the same plot, as their difference can point to possible penetration or strong disturbance of the membrane by the compounds. However, the question of how the presence of sterol changes membrane properties with respect to its interaction with different surfactants is very interesting. Thus, we combined the data of the Figure 3 to present DOPC vs. DOPC/Chol curves in the same graph (Figure 4).

From the plots, it follows that addition of cholesterol increases adsorption of Kor105 onto the membrane, as determined by both IFC and nonactin conductance methods (Figure 4A,B). On the contrary, cholesterol slightly hampers adsorption of SDS, although this conclusion is supported reliably only by IFC measurements (Figure 4E). Taking into account the experimental errors, cholesterol does not influence adsorption of BAC (Figure 4C,D).

In summary, the experiments on BLMs demonstrated that all three tested compounds, Kor105, BAC, and SDS, adsorb onto the membranes of both DOPC and DOPC/Chol 3:2 compositions, yielding substantial boundary potentials. For the compounds, the boundary potentials determined by IFC and nonactin conductance methods were very similar, indicating the ions neither passed through the membrane nor crucially disturbed the monolayer dipoles. Cholesterol substantially increased adsorption of Kor105, slightly hampered adsorption of SDS, and had almost no effect on adsorption of BAC. In the presence of cholesterol, all three tested ions yielded almost the same magnitude of the boundary potential difference within experimental error.

### 3.2. MD Modeling of Membranes with Incorporated Hydrophobic Ions

To test the preliminary conclusions on the interaction of hydrophobic ions with model BLMs, we attempted molecular modeling. Membranes of 12 different compositions were modeled: (1) 200 DOPC, one Kor105 (lowKorDOPC); (2) 120 DOPC, 80 Chol, one Kor105 (lowKorCholDOPC); (3) 202 DOPC, 86 Kor105 (highKorDOPC); (4) 122 DOPC, 80 Chol, 86 Kor105 (highKorCholDOPC); (5) 200 DOPC, one BAC (lowBacDOPC); (6) 120 DOPC, 80 Chol, one BAC lowBacCholDOPC; (7) 202 DOPC, 86 BAC (highBacDOPC); (8) 122 DOPC, 80 Chol, 86 BAC (highBacCholDOPC); (9) 200 DOPC, one SDS (lowSDSDOPC); (10) 120 DOPC, 80 Chol, one SDS (lowSDSCholDOPC); (11) 202 DOPC, 86 SDS (highSDSDOPC); (12) 120 DOPC, 80 Chol, 86 SDS (highSDSCholDOPC). Each simulation box was filled with at least 10,000 water molecules. The bilayers were analyzed through a 500-ns MD run at constant temperature and pressure.

**Figure 5:**
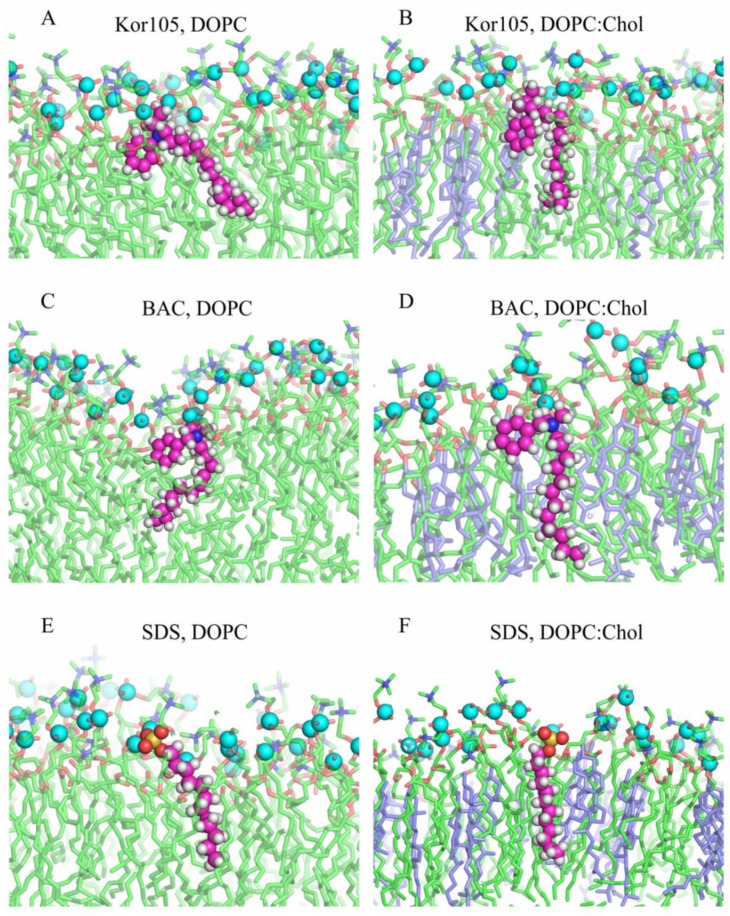
Representative snapshots of Kor105 (**A**,**B)**, BAC (**C**,**D**), and SDS (**E**,**F**) conformations in lipid membranes formed from DOPC (**A**,**C**,**E**) and the DOPC/Chol 3:2 mixture (**B**,**D**,**F**). C, H, N, and S atoms of the ions are shown as magenta, white, blue, and yellow spheres, respectively. Lipids are shown in stick representation, with phosphorus atoms indicated by cyan spheres. DOPC tails are shown as green sticks, cholesterol as blue sticks, O atoms as red sticks, and N atoms of DOPC as blue sticks.

It appeared that, in lipid membranes, SDS conformation was linear with sulfate located in the region of the lipid phosphate groups. Both BAC and Kor105 adopted a kinked conformation. In this conformation, the N^+^ atom was located in the region of the lipid phosphate groups, the alkyl tail was buried in the membrane interior, and the aromatic ring was positioned in the region of lipid ester bonds, i.e., at the membrane polar–hydrophobic interface (Figure 5).

**Figure 6:**
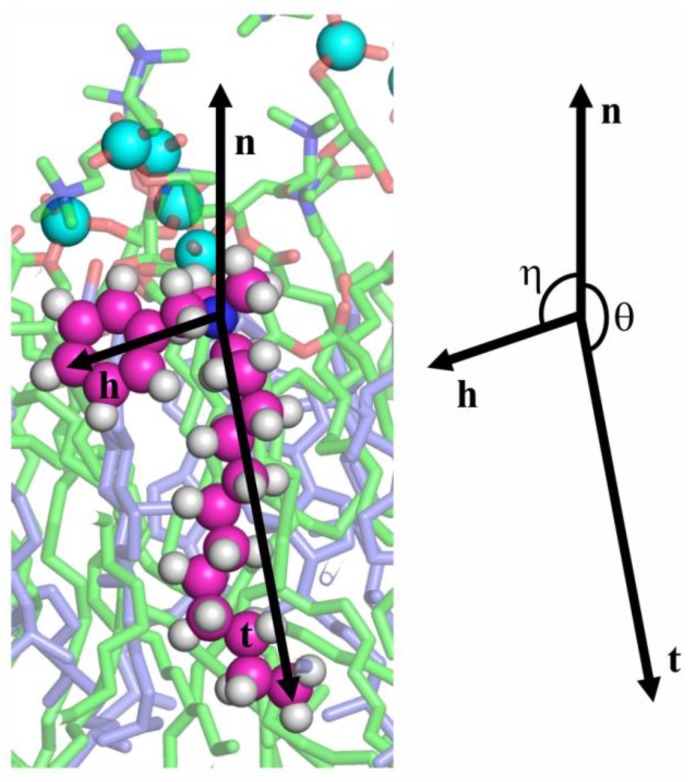
Characterization of hydrophobic ion orientation in a lipid membrane. C, H, and N atoms of the hydrophobic ion are shown as magenta, white, and blue spheres, respectively. Lipids are shown in stick representation, with phosphorus atoms indicated by cyan spheres. DOPC tails are shown as green sticks, cholesterol as blue sticks, O atoms as red sticks, and N atoms of DOPC as blue sticks. **n** is the unit normal vector to the membrane surface, **h** is the vector directed from the N^+^-atom (blue sphere) toward the C-atom in the *para* position of the aromatic ring, and **t** is the vector directed from the N^+^-atom (or from the S-atom of SDS) toward the terminal C-atom in the alkyl tail in the hydrophobic ion. The orientation of the hydrophobic ion is characterized by the angle *η* between **n** and **h** and the angle *θ* between **n** and **t** (the scheme on the right).

Let us denote the vector directed from the N^+^-atom toward the C-atom in the *para* position of the aromatic ring in the hydrophobic ion as **h**, the vector directed from the N^+^-atom or S-atom toward the terminal C-atom in the alkyl tail in the hydrophobic ion as **t**, and the vector of the unit external normal to the membrane plane as **n** (Figure 6).

The orientation of hydrophobic ions was characterized by two angles: (1) *η*, the angle between **n** and **h**; (2) *θ*, the angle between **n** and **t** (Figure 6). From the MD trajectories, the distributions of the angles *η* and *θ* were analyzed both for the single molecules of the hydrophobic ions (systems lowKorDOPC, lowKorCholDOPC, lowBacDOPC, lowBacCholDOPC, lowSDSDOPC, lowSDSCholDOPC) and for their ensembles (systems highKorDOPC, highKorCholDOPC, highBacDOPC, highBacCholDOPC, highSDSDOPC, highSDSCholDOPC).

**Figure 7:**
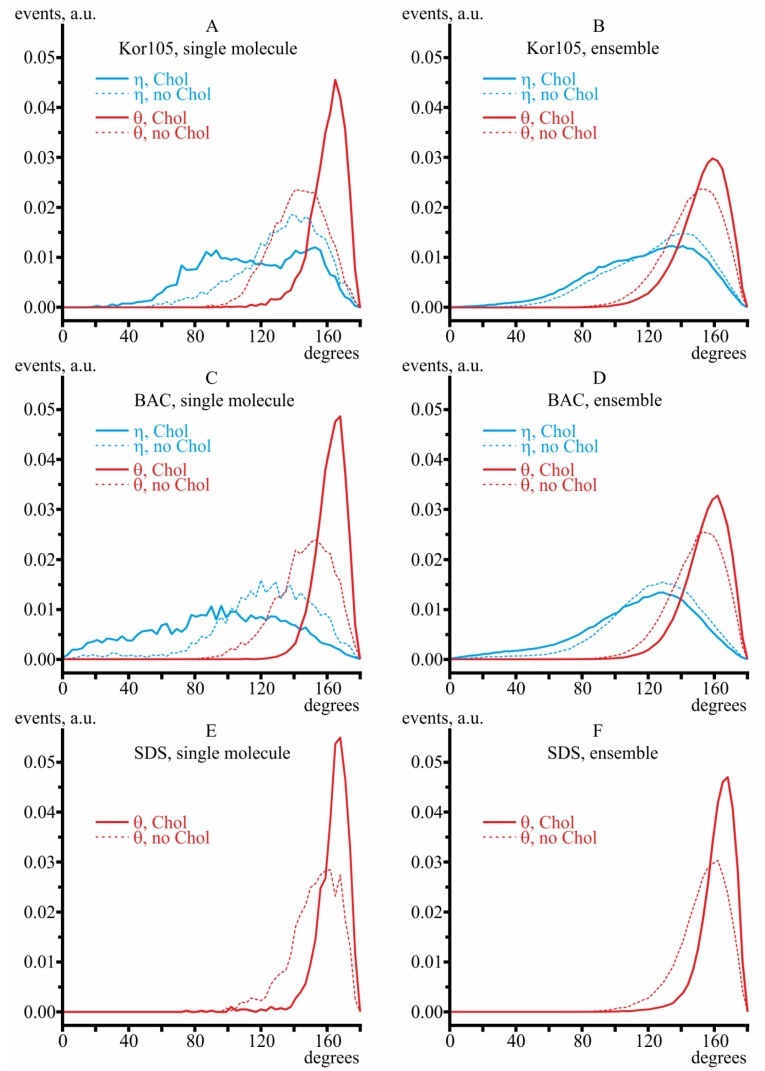
The density of the distribution of angles *η* (blue lines) and *θ* (red lines) for Kor105 (**A**,**B**) and BAC (**C**,**D**). The density of the distribution of the angle *θ* (red lines) for SDS is shown in (**E**,**F**). The distributions were calculated for the case of a single molecule of hydrophobic ions in the membrane (**A**, **C**, and **E**, systems lowKorDOPC, lowKorCholDOPC, lowBacDOPC, lowBacCholDOPC, lowSDSDOPC, lowSDSCholDOPC) and for the case of a high concentration of hydrophobic ions in the membrane (**B**, **D**, and **F**, systems highKorDOPC, highKorCholDOPC, highBacDOPC, highBacCholDOPC, highSDSDOPC, highSDSCholDOPC); see Section 2.2 for more details on membrane composition. In all panels, thin dashed lines correspond to DOPC membranes; thick solid lines correspond to DOPC/Chol 3:2 membranes.

In pure DOPC membranes, the alkyl tails of both hydrophobic ions were highly dynamic; they were irregularly bent, and the chain was randomly oriented with respect to the average normal to the membrane surface (Figure 5A,C,E). In the presence of cholesterol, the tails were more ordered; they were almost straight and oriented nearly perpendicular to the membrane surface (Figure 5B,D,F). The density of the distribution of angles *η* and *θ* is shown in Figure 7 for both single molecules of hydrophobic ions (systems lowKorDOPC, lowKorCholDOPC, lowBacDOPC, lowBacCholDOPC, lowSDSDOPC, lowSDSCholDOPC) and their ensembles (systems highKorDOPC, highKorCholDOPC, highBacDOPC, highBacCholDOPC, highSDSDOPC, highSDSCholDOPC). As SDS lacks the aromatic ring, only the distribution of *θ* angle was plotted for this ion (Figure 7E,F).

In cholesterol-free membranes, the angle *η* between the vector normal to the membrane surface and the plane of the aromatic ring was approximately 130° in all modeled systems, meaning that the molecule of the hydrophobic ion in such a membrane is strongly bent around its N^+^ atom (*θ* ≈ 150°; thus, the angle between **h** and **t** vectors is approximately *θ* − *η* ≈ 20°), and the aromatic ring is buried deeply into the hydrophobic core of the lipid monolayer. From the plots, especially those built for single molecules, it follows that cholesterol on average decreases *η* and increases *θ*, which means that the hydrophobic ion molecule partly unbends in the presence of cholesterol, as the difference (*θ* − *η*) increases. The ion alkyl tail aligns with the average direction of the lipid tails, which follows from the increase of *θ*.

Note that, for a single BAC molecule in both cholesterol-free and cholesterol-containing membranes, the distributions of *η* and *θ* angles are very wide (Figure 7C). However, at high BAC concentrations, the distributions become relatively narrow, meaning that the order of the membrane is increased upon incorporation of more BAC molecules (Figure 7D). For Kor105, the distributions of *η* and *θ* angles have comparable widths for both the single molecule and the ensemble of the hydrophobic ion (compare Figure 7A,B). For SDS the distributions of *θ* angle are relatively narrow in both DOPC and DOPC/Chol membranes, as well as for a single SDS molecule and the ensemble (Figure 7E,F).

**Figure 8:**
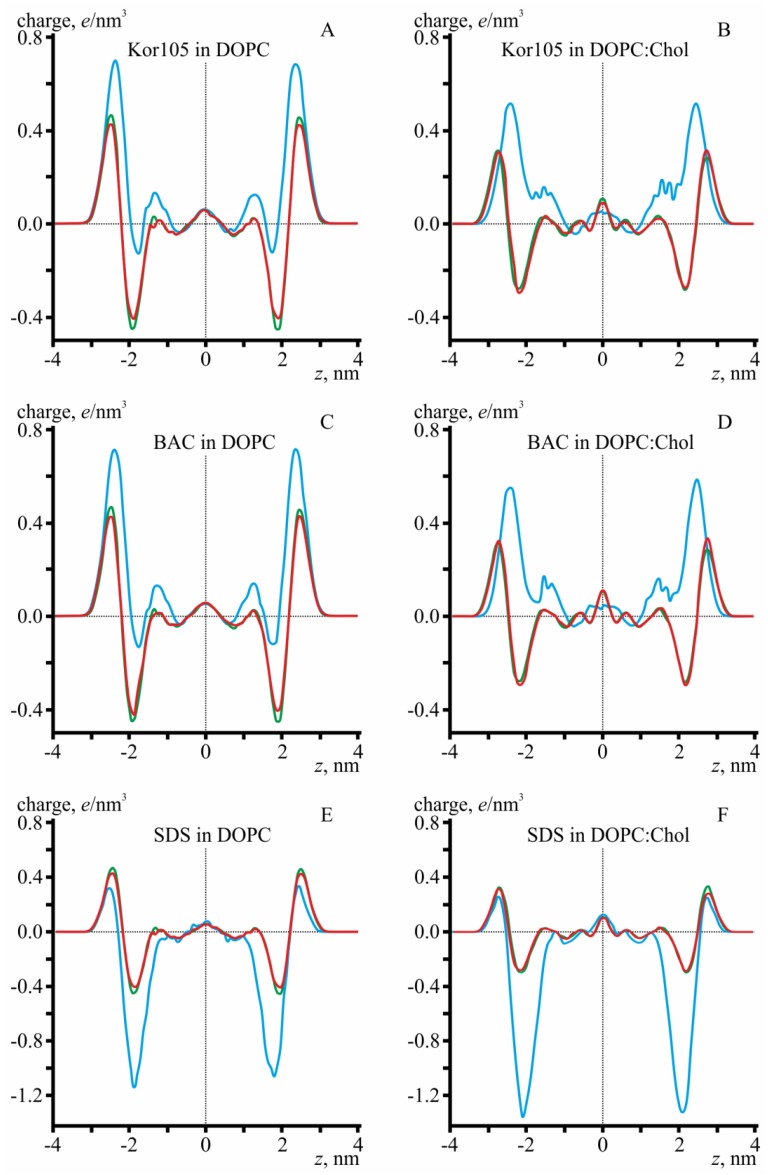
Distribution of the electrical charge density along the *Oz* axis (perpendicular to the membrane plane). Green curves are lipid membranes without hydrophobic ions, red curves are single molecules of the hydrophobic ion located in the upper monolayer (positive *z*), and blue curves show high concentrations of hydrophobic ions, evenly distributed between the lipid monolayers. *z* = 0 corresponds to the monolayer interface (middle of the membrane). **A**—Kor105 in the DOPC membrane; **B**—Kor105 in the DOPC/Chol 3:2 membrane; **C**—BAC in the DOPC membrane; **D**—BAC in the DOPC/Chol 3:2 membrane; **E**—SDS in the DOPC membrane; **F**—SDS in the DOPC/Chol 3:2 membrane.

To analyze the depth of incorporation of hydrophobic ions into the membranes, we considered the distribution of electrical charge density along the direction of the normal vector to the membrane surface, **n**, i.e., along the *Oz* axis (Figure 8). In the cholesterol-free membrane, the positive charges of BAC and Kor105 are located predominantly in the region between the phosphorus and nitrogen of DOPC, independent of the ion type (Figure 8A,C). The addition of cholesterol results in a smaller average charge density of lipids, as cholesterol does not bear any charge and merely dilutes the charges located on nitrogen and phosphorus atoms of DOPC, leading to smaller peaks (compare the amplitudes of the peaks of the green curves in Figure 8A,C,E, as well as in Figure 8B,D,F). Besides, cholesterol addition leads to an increase in the membrane thickness, as the nitrogen and phosphorus atoms of DOPC both shift by approximately 0.4 nm in the direction from the membrane center (compare the location of the positive and negative peaks of the green curves in Figure 8A,C,E, as well as in Figure 8B,D,F). In particular, the negative peak corresponding to phosphorus atoms of DOPC shifts from *z* = ±1.8 nm to *z* = ±2.2 nm. Interestingly, the position of the positive peak, corresponding to the N^+^ atom of the hydrophobic ion, does not change upon cholesterol addition (compare the position of the positive peaks of the blue curves in Figure 8A,C, as well as in Figure 8B,D). Although charged atoms of DOPC do shift, the N^+^ atom remains at *z* = ±2.5 nm for both Kor105 and BAC, independent of the cholesterol content in the membrane. The effective result is that, in cholesterol-containing membranes, the hydrophobic ion is buried deeper by approximately 0.4 nm into the lipid monolayer compared to the case of membranes formed from pure DOPC. In cholesterol-free membranes, the positive peak is relatively high in the case of high concentrations of hydrophobic ions; nevertheless, the zone of negative electric charge is still detectable at *z* = ±1.8 nm (Figure 8A,C). In cholesterol-containing membranes, the positive peak is lower due to dilution of electric charges by the molecules of electrically neutral sterol, but the negative peaks at *z* = ±1.8 nm completely disappear (Figure 8B,D).

Notably, the MD simulations suggested very similar behavior of both BAC and Kor105 in cholesterol-containing membranes (Figure 7 and Figure 8). However, the data presented in Figure 3 and Figure 4 clearly indicate the difference between the effects of cholesterol on BAC and Kor105 interaction with membranes (Figure 4, A vs. C and B vs. D). Most likely, this difference is due to the fact that, in Kor105, the aromatic ring is directly linked to nitrogen, which makes the ring less mobile, as compared to BAC. This means that Kor105, being relatively more rigid, disturbs the lipid monolayer more strongly than BAC. This disturbance is more pronounced in the membranes rich in cholesterol, as cholesterol orders the Kor105 hydrophobic tail, thus imposing a stricter orientation of the aromatic ring.

Quite surprisingly, the negatively charged sulfate group of SDS is located in the region of phosphorus atoms of lipids (Figure 8E,F). Such localization appears to be independent of cholesterol content. Addition of cholesterol shifts the negative peak of phosphorus atoms of the lipids from the center of the membrane (compare locations of negative peaks of green curves in Figure 8E,F). The negative peak of SDS sulfate group shifts in the same direction by the same distance, from *z* = ±1.8 nm to *z* = ±2.2 nm (compare locations of negative peaks of blue curves in Figure 8E,F). This behavior is the opposite to that demonstrated by BAC and Kor105; the location of their nitrogen atoms with respect to membrane center did not change upon addition of cholesterol, although phosphorus atoms of lipids shifted. The behavior of SDS can be explained by it generally preferring to bury its sulfate in the region of positively charged choline groups of the lipids. However, this would place SDS hydrophobic tails near to the polar region of the lipid phosphate groups, leading to a high energy penalty. Burying the SDS deeper into the lipid monolayer would lead to contact of lipid hydrophobic tails with electric charges of the SDS sulfate group. It appears that, in such a situation, it is energetically favorable to minimize the contact between polar and hydrophobic groups, despite the electrostatic repulsion between the neighboring sulfate of SDS and phosphate groups of the lipids.

**Figure 9:**
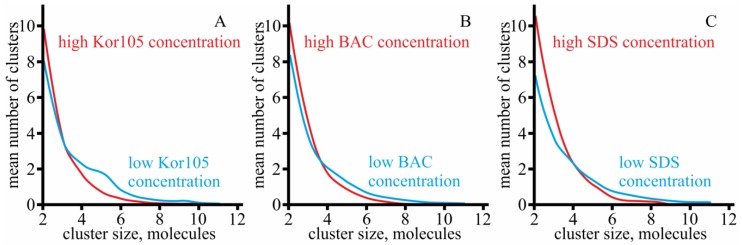
Size distribution of cholesterol clusters for **A**—Kor105 ion, lowKorCholDOPC, and highKorCholDOPC systems; **B**—BAC ion, lowBacCholDOPC, and highBacCholDOPC systems; and **C**—SDS ion, lowSDSCholDOPC, and highSDSCholDOPC systems. Blue curves—low concentration of the ion; red curves—high concentration of the ion.

For all modeled systems, the molecules were distributed evenly between the monolayers, and laterally randomly along each monolayer. However, in the course of the MD runs, we observed that cholesterol formed small clusters. We analyzed the size distribution of cholesterol clusters using the last 100 ns of the trajectories for the analysis; the number of clusters was calculated at each integration step. The cholesterol molecules were considered as clustered if the distance between their O atoms was smaller than 0.7 nm. The size distribution of cholesterol clusters is presented in Figure 9. It was found that the size distribution depended on the concentration of the ion; higher concentrations yielded smaller clusters. This effect can be explained by favorable interaction of saturated alkyl chains of the ions with cholesterol, leading to disruption of cholesterol clusters and heterodimerization of the ion and cholesterol molecules. However, simple dilution of cholesterol by the ions cannot be excluded. Each modeled system contained approximately the same number of cholesterol molecules. Thus, for a high concentration of hydrophobic ions, the molar fraction of cholesterol was lower compared to the membranes with a low concentration of hydrophobic ions. Such a dilution might be the main reason responsible for the smaller size of the clusters at high concentrations of hydrophobic ions.

**Figure 10:**
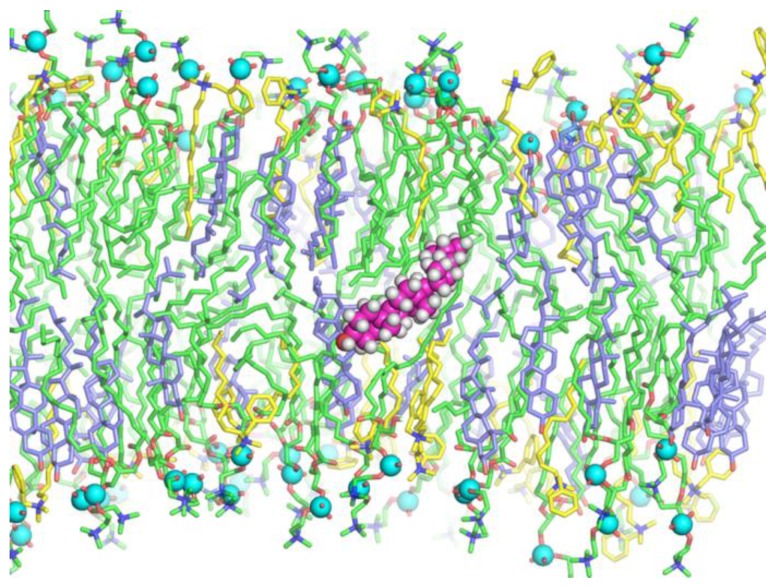
Plunging of cholesterol molecules into the bilayer for the highBacCholDOPC system. The plunging was observed twice during the 500-ns trajectory. Cholesterol molecules laid at the monolayer interface for 4 ns and 16 ns. C, H, and O atoms of the plunged cholesterol molecules are shown as magenta, white, and red spheres, respectively. Lipids are shown in stick representation, with phosphorus atoms indicated by cyan spheres. DOPC tails are shown as green sticks, cholesterol as blue sticks, O atoms as red sticks, and N atoms as blue sticks. BAC molecules are shown as yellow sticks.

Generally, no redistribution of ions (BAC, Kor105, or SDS) between the lipid monolayers was observed during 500 ns of each simulation trajectory. The flip-flop of the cholesterol was also absent with some exceptions. For the system highBacCholDOPC composed of 122 DOPC, 80 Chol, and 86 BAC molecules, cholesterol molecules twice plunged into the bilayer, laid at the monolayer interface for 4 ns and 16 ns (Figure 10), and returned to the same monolayer. For the system highKorCholDOPC composed of 122 DOPC, 80 Chol, and 86 Kor105 molecules, cholesterol molecules several times plunged into the bilayer, but did not reach the monolayer interface, and immediately returned to the same monolayer.

MD modeling showed that the presence of cholesterol increases the ordering of the lipid membrane, leading to a greater thickness of the lipid bilayer. The increased bilayer thickness does not change the position of the N^+^ atom of BAC or Kor105 with respect to the monolayer interface. Thus, cholesterol addition results in effectively deeper penetration of BAC and Kor105 into the lipid monolayer as related to the positions of the lipid headgroups. On the contrary, for SDS, it was shown that, upon addition of cholesterol, the location of the SDS sulfate group follows the position of phosphates of the lipids; as the lipid monolayer becomes thicker, the sulfate group also shifts from the bilayer center, always being co-localized with the phosphates. The MD simulations showed a principal difference between the interactions of SDS and the cationic surfactants with the membranes. For all considered ions, the positions of the charged groups are close to the negatively charged phosphate groups of DOPC. This means that, due to electrostatic interactions, the cationic surfactants are significantly more membranophilic than anionic SDS. Thus, although the tested model membranes are electrically neutral, their interaction with hydrophobic ions depends on the sign of the charge of the ion. Another principal difference comes from the fact that, while the SDS molecule is essentially linear, the BAC and Kor105 molecules are not; their aromatic heads are wider that the tails. For this reason, the membrane disturbance caused by the cationic surfactants is sensitive to cholesterol; this neutral lipid dampens the surfactant-induced misalignment of the hydrophobic tails of DOPC. On the contrary, within the range of tested concentrations, SDS does not alter the packaging of the hydrophobic tails, which explains why cholesterol presence does not significantly affect SDS–membrane interaction, as shown by the experiments on BLM.

### 3.3. Experiments on Yeast Cells with Deletion of PM Sterol Transporters

Our results suggested that the sterol content of the PM strongly influences the effects of benzalkonium and Kor105 on cell physiology. To test this hypothesis, we used yeast cells harboring mutations in *Lam* genes, which encode PM sterol transporters. Lam1–4 proteins were shown to transport ergosterol, the main yeast sterol, from the PM to the ER (reviewed in References [22,38]). Their deletions are believed to affect either the ergosterol content of the PM, its distribution within the PM, or both [20,21]. There are several lines of evidence pointing to the *Lam* mutations leading to an increase in PM ergosterol. Firstly, Lams are passive sterol transporters and the PM is the richest cellular compartment in terms of sterol concentration. Secondly, delta-Lams are sensitive to amphotericin B [20]. Amphotericin B is a drug which forms ion channels in ergosterol-containing but not in ergosterol-free plasma membranes [39]. Thus, increased amphotericin B sensitivity can be attributed to an increased ergosterol content. Thirdly, it was demonstrated that the deletion of mammalian *Lam* homologs leads to a decreased flow of cholesterol from the PM inside the cells [23].

**Figure 11:**
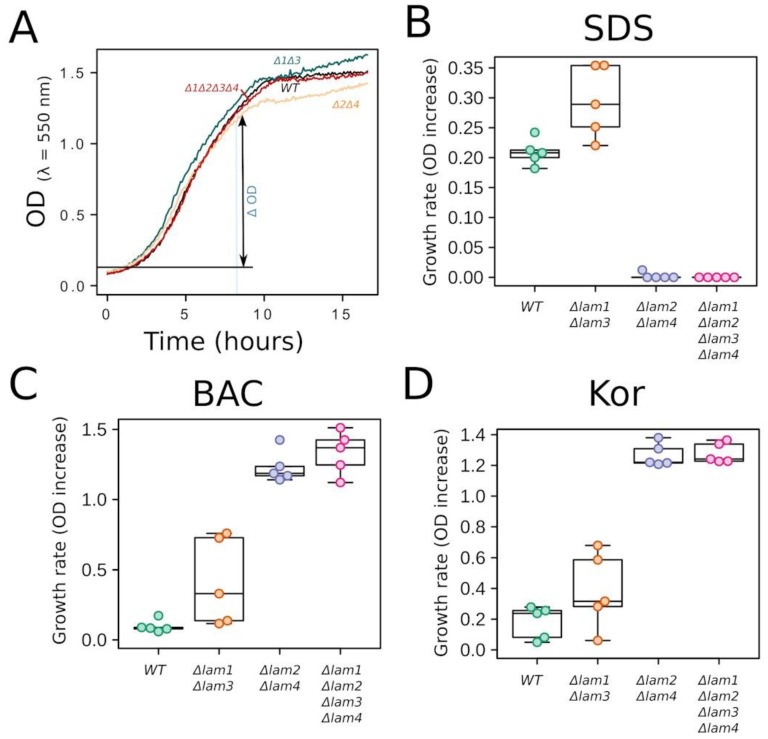
*△Lam2△Lam4* double-gene deletion decreases the resistance to SDS and increases the resistance to BAC and Kor105. (**A**) Representative growth curves of the wild-type and *Lam* deletion strains. (**B**–**D**) Growth rate of yeast mutants in the presence of negatively charged surfactant SDS, 0.01% (**B**) and positively charged surfactants BAC, 5 µg/mL (**C**) and Kor105, 2.5 µg/mL (**D**). To quantify the growth rates, we measured an increase in the yeast suspension optical density (OD) from 1 to 9 h of growth.

We constructed three *Lam* deletion mutants: *△Lam1△Lam3*, *△Lam2△Lam4*, and quadruple *△Lam1△Lam2△Lam3△Lam4* mutants. Firstly, we found that deletions do not strongly affect the growth rate of the cells, at least when the cells are grown in a standard rich medium (Figure 11A).

Next, we compared the resistances of the control strain and the deletion mutants to SDS, benzalkonium, and Kor105. The cells were grown in rich liquid medium in the presence of the indicated concentrations of the surfactants. The drug resistance was estimated by measuring the growth rates (Figure 11). The double deletion *△Lam1△Lam3* had no significant effect on the resistances (Figure 11B–D). Furthermore, the *△Lam2△Lam4* and *△Lam1△Lam2△Lam3△Lam4* deletion strains were more sensitive to SDS than the control strain (Figure 11B). In contrast, these two mutant strains were less sensitive to the cationic surfactants than the control (Figure 11C,D).

Together, the data presented in Figure 11 indicate that even relatively minor alterations (most likely an increase) in the PM sterol can cause a major change in the resistance to the surfactants. Also, our data show that the same alterations in the PM sterol content can change the resistance to SDS and the cationic surfactants in opposite directions. Our finding that *△Lam* mutants are more sensitive to SDS than the wild type is not surprising; deletion mutations tend to make the cells generally weaker and, thus, more sensitive to a variety of poisonous substances. For the same reason, an increase in the resistance caused by even the quadruple mutation requires a more specific explanation. We reason that an increase in ergosterol levels in the PMs of *△Lam* mutants can explain their increased resistance to BAC and Kor105. Indeed, the MD showed that cholesterol reduces the disturbance to the lipid packaging caused by these surfactants (but not that caused by SDS, which seems to be not influenced by sterols at all).

## 4. Conclusions

In our study, we used three independent experimental approaches to study the effects of sterol presence in the membranes on their interaction with surfactants.

Firstly, we showed that SDS and benzalkoniuminduced changes in the BLM surface potential were almost identical for cholesterol-free and cholesterol-rich membranes. In contrast, sterol addition drastically changed the mode of interaction of a novel benzalkonium-like compound, Kor105.

Secondly, we modeled the interaction of SDS, benzalkonium and Kor105 with the membrane using MD simulations. The modeling showed that the charged group of benzalkonium penetrated deeper than the layer of the negatively charged phosphate groups of the sterol-rich monolayer. According to the simulations, in the case of the sterol-free membrane, the charged groups of benzalkonium did not penetrate as deeply. Intriguingly, the experiments on the planar bilayer membrane showed that, while benzalkonium and Kor105 interacted in a similar way with the sterol-free membranes, their interactions with the cholesterol-rich membranes were different due to the more rigid structure of the latter compound. Kor105 changed the surface potential of the sterol-free membrane to a lesser extent than the potential of the sterol-rich membrane. Additionally, the modeling showed that cholesterol suppressed the disturbance of the lipid packaging caused by BAC and Kor105, while the interaction of SDS with the membranes was not sensitive to cholesterol.

Thirdly, the experiments on yeast showed that the deletions of the PM sterol transporters differently affect the resistance of the cells to SDS versus the cationic surfactants. These experiments exemplify that a minor alteration in sterol composition can change the membrane resistance to surfactants, whereas the direction of the effect depends on the surfactant charge.

Taken together, all three experimental approaches indicate that alterations in the sterol content differentially affect the interactions of SDS vs. BAC and Kor105 with the membranes. Additionally, our data suggest that sterols in the membranes increase the resistance to cationic surfactants to a higher extent than anionic ones. While the increased sensitivity of *△Lam* mutants to SDS could be due to general harm to the cell physiology caused by the deletions, the increase in the resistance to the cationic surfactants is harder to explain. Our data, together with the previous observations, suggest that higher ergosterol in the PMs of *△Lam* mutants reduces the membrane disturbance caused by BAC and Kor105. Finally, we speculate that the experiments on the planar bilayer membrane might have a practical application. Possibly, they can serve as a screening platform for chemicals that do not affect PMs of human cells and, at the same time, alter the properties of the PMs of pathogenic microorganisms. Such screening might be instrumental in the search for novel antiseptics/antibiotics [40,41,42].
